# Registration—Proposed Law

**Published:** 1873-12

**Authors:** 


We desire to call the attention of the profession in this State to the action of our State Association, at its last meeting, upon the form of a Bill for the registration of deaths, marriages and births, introduced by Dr. C. B. Nottingham, of Macon, which was proposed for adoption by the Legislature of the State, at its coming January session. We trust that physicians of the State will call the attention of members of the Legislature to this Bill and urge its passage.

